# Investigation of chemotherapy-induced brain structural alterations in breast cancer patients with generalized q-sampling MRI and graph theoretical analysis

**DOI:** 10.1186/s12885-018-5113-z

**Published:** 2018-12-04

**Authors:** Tsung-Yuan Li, Vincent Chin-Hung Chen, Dah-Cherng Yeh, Shu-Ling Huang, Cheng-Nan Chen, Jyh-Wen Chai, Clayton Chi-Chang Chen, Jun-Cheng Weng

**Affiliations:** 1 Department of Radiology, Taichung Veterans General Hospital, Taichung, Taiwan; 2 School of Medicine, Chang Gung University, Taoyuan, Taiwan; 3 Department of Psychiatry, Chang Gung Memorial Hospital, Chiayi, Taiwan; 4 Breast Medical Center, Cheng Ching Hospital Chung Kang Branch, Taichung, Taiwan; 5 Department of Psychology, Chung Shan Medical University, Taichung, Taiwan; 6 College of Medicine, China Medical University, Taichung, Taiwan; 7 Department of Medical Education, Taichung Veterans General Hospital, Taichung, Taiwan; 8 Department of Medical Imaging and Radiological Sciences, Chang Gung University, No. 259, Wenhua 1st Rd., Guishan Dist., Taoyuan City, 33302 Taiwan

**Keywords:** Breast cancer, Chemotherapy, Generalized q-sampling imaging, Voxel-based statistical analysis, Multiple regression analysis, Graph theoretical analysis, Network-based statistical analysis

## Abstract

**Background:**

Breast neoplasms are the most common cancer among women in Taiwan. Cognitive deficits are common complications of breast cancer survivors treated with chemotherapy. The most frequently observed disorders involve executive function and memory impairment. With improvements in tumor intervention and the consequent increase in the number of cancer survivors, the quality of life of patients has become an important issue. We are interested in the early effects of chemotherapy on the brain structures of patients. In addition, generalized q-sampling imaging (GQI), a wide range of q-space datasets for a more accurate and sophisticated diffusion MR approach, was first used in this topic.

**Methods:**

As diffusion tensor imaging (DTI) is associated with restrictions in the resolution of crossing fibers, we attempted to use GQI, which can overcome these difficulties and is advantageous over DTI for tractography of the crossing fibers. This cross-sectional study included two groups: breast cancer survivors who had completed their chemotherapy (*n* = 19) and healthy controls (*n* = 20). All participants underwent diffusion MRI exams and neuropsychological assessments. We included four parts in our image analysis, i.e., voxel-based statistical analysis, multiple regression analysis, graph theoretical analysis and network-based statistical analysis.

**Results:**

The results from the voxel-based statistical analysis showed significantly lower GFA and NQA values in the breast cancer group than those in the control group. We found significant positive correlations between the FACT-Cog and GQI indices. In the graph theoretical analysis, the breast cancer group demonstrated significantly longer characteristic path length. Adjuvant chemotherapy affected the integrity of white matter and resulted in poor cognitive performance, as indicated by the correlations between the neuropsychological assessment scales and the GQI indices. In addition, it was found that the characteristic path lengths in the breast cancer group increased, indicating that the brain network integration became worse.

**Conclusions:**

Our study demonstrated alterations in structural brain networks and associated neuropsychological deficits among breast cancer survivors.

## Background

The most common cancer among women in Taiwan is breast neoplasms. There are more than 10,000 women suffering from breast cancer, and nearly 2000 women die of breast cancer annually [1, 2]. The stage of breast cancer is based on the tumor size, the axillary lymph nodes that are involved, and distant metastasis. If breast cancer can be diagnosed and treated earlier, the five-year survival rate would be projected to increase to a maximum of 90%. There are various treatments for breast cancer, including traditional surgery, adjuvant chemotherapy, radiation therapy, targeted therapy and hormonal therapy.

### Cognitive function in breast cancer survivors

Breast cancer is a common cause of mortality; however, treatments have improved, and consequently, the interest in the quality of life and function among survivors has increased. Therefore, depression, anxiety and psychiatric symptoms among breast cancer survivors need to be investigated. Breast cancer patients receive different treatments based on the size of the tumor, and these treatments often result in physical or cognitive deficits in patients. A range of 15 to 50% of patients with malignant tumors show persistent cognitive impairments after chemotherapy [3]. Many studies have also reported that breast cancer patients often appear to develop the phenomenon of chemo-brain after chemotherapy. In this condition, patients often complain of problems regarding memory, concentration, multiple operation, processing speed, and word retrieval [4, 5]. This cognitive impairment in patients can affect social relationships and even work performance [5].

One study tracked changes in the cognitive function of three groups, which included breast cancer patients with chemotherapy combined with radiotherapy, breast cancer patients with radiotherapy only, and a control group, over 3 years. The breast cancer patients treated with chemotherapy and radiotherapy (CTRT; *n* = 62) or radiotherapy only (RT; *n* = 67) completed neuropsychological assessments 6 months after completing treatment and then again 36 months later. The control group (*n* = 184) was assessed over a similar interval. There was also a significant difference in executive function (*p* = 0.006) among the three groups. This difference indicated that the control group performed better than the CTRT and RT groups [6].

### Diffusion magnetic resonance imaging of the brain

With the improvements in tumor intervention and the increase in the number of cancer survivors, the cognition of patients has become an important issue for patients, physicians and researchers. One study evaluated the long-term effect of chemotherapy on brain microstructural integrity by comparing the brains of chemotherapy-exposed breast cancer survivors to those of healthy women. There were two groups of participants: 187 breast cancer survivors treated with CMF (cyclophosphamide, methotrexate, and 5-flourouracil) and 374 age-matched healthy women. Diffusion tensor imaging (DTI) was analyzed with tract-based spatial statistics. In addition, the authors used linear regression analysis to explore the impact of the length of time after chemotherapy. The results showed that the length of time after chemotherapy was inversely associated with fractional anisotropy (FA), mean diffusivity (MD) and radial diffusivity (RD) among breast cancer survivors. The authors reported that adjuvant chemotherapy had an adverse effect on the integrity of the white matter microstructure of breast cancer survivors who had survived more than 20 years on average [7].

In this study, we evaluated the early effects of chemotherapy on the brain structures of patients. Since diffusion tensor imaging is associated with restrictions in the resolution of crossing fibers, we tried to use generalized q-sampling imaging (GQI), which can overcome these difficulties and is advantageous over DTI for the tractography of crossing fibers [8].

## Methods

### Participants

This study was a cross-sectional study, and participants were recruited from the department of breast surgery of Taichung Veterans General Hospital. The study included 19 women with a history of breast cancer (stage I-IIIA) who had completed their primary chemotherapy less than 6 months before study entry and were currently without evidence of active cancer. There were 4 patients received radiation therapy, and 1 patient received hormone treatment. The number of menopausal women in the patients and controls were 5:5. The average age of the breast cancer survivors was 43.8± 6.4 years. The only chemotherapeutic drugs used by the patients were taxotere and epirubicin. Another 20 healthy women aged 50.1± 2.5 years served as the control group. All participants underwent magnetic resonance imaging (MRI) examinations on a 1.5 T scanner (Aera, Siemens, Germany) and neuropsychological assessments. The clinical characteristics and neuropsychological assessments are shown in Table 1.

**Table 1:** Group differences in clinical characteristics and neuropsychological assessment

Characteristic	Patients Treated After Chemotherapy (*n* = 19)		Healthy Controls (*n* = 20)		*P*
	Mean or Count	SD	Mean or Count	SD	
Age (years)	43.8	6.4	50.1	2.5	0.001
Education (years)	13.9	2.2	13.3	2.3	0.435
Breast cancer stage (0, I, II, III, IV)	(0, 2, 14, 3, 0)	N/A	N/A	N/A	N/A
Chemotherapeutic drugs (Taxotere and Epirubicin)	19	N/A	N/A	N/A	N/A
Radiation therapy	4	N/A	N/A	N/A	N/A
Hormonal treatment	1	N/A	N/A	N/A	N/A
Menopausal	5	N/A	5	N/A	N/A
MMSE	28	1.283	28.316	1.453	0.508
CAMS-R	33.882	4.471	33.895	3.972	0.993
IES-R	15.941	24.055	7.079	10.498	0.187
Hospital Anxiety and Depression Scale (HADS)					
Anxiety	7.118	5.075	6.474	3.485	0.666
Depression	3.941	3.842	5.474	2.702	0.184
Functional Assessment of Cancer Therapy-Cognitive Function (FACT-Cog)					
Perceived cognitive impairments	52.588	10.319	58.789	7.522	0.052
Comments from others	13.765	2.438	14.158	1.954	0.606
Perceived cognitive abilities	17.882	4.523	18	5.4	0.946
Impact on quality of life	11.765	3.557	14.211	1.989	0.023

The inclusion criteria were as follows: breast cancer survivors (within 6 months after chemotherapy) 20–55 years of age and healthy female 20–55 years of age. There were no other cancer types present in the breast cancer survivors other than breast cancer. If the participants were diagnosed with psychiatric, neurologic, or comorbid medical conditions that are known to affect cognitive function, they were excluded.

### Procedure

This study was approved by the Institutional Review Board at Taichung Veterans General Hospital. The review number was SF14185A. Participants were recruited from the department of breast surgery of Taichung Veterans General Hospital. The research assistant explained the research proposal to participants so that the participants could understand the research purpose, process and both their rights and interests. Clinical physicians assessed the physiological status of each participant to ensure she could participate in the MRI examination. All participants provided written informed consent before the examination. A clinical psychologist performed the neuropsychological assessment, and a radiologic technologist performed the subsequent MRI examination. The overall process took approximately 90 min.

### Neuropsychological assessments

We designed the questionnaire to understand the basic information of the participants and used objective and subjective psychological tests to evaluate the cognitive function, emotion, mindfulness and psychological trauma of the participants. The neuropsychological tests included the Mini-Mental State Examination (MMSE), Functional Assessment of Cancer Therapy-Cognitive Function (FACT-Cog), Hospital Anxiety and Depression Scale (HADS), Impact of Event Scale-Revised (IES-R), and Cognitive and Affective Mindfulness Scale-Revised (CAMS-R). All statistics were performed with Microsoft Excel 2010. The results of the neuropsychological assessments are shown in Table 1.

### Diffusion imaging parameters

For diffusion imaging, we performed a single-shot, diffusion-weighted spin echo-planar imaging sequence with the following parameters: magnetic field strength = 1.5 Tesla, repetition time = 7200 msec, echo time = 107 msec, field of view = 256 mm, matrix = 128 × 128, slice thickness = 4 mm, resolution = 2 × 2 × 4 mm^3^, b-values = 0, 1000, and 2000 s/mm^2^ in 129 noncollinear directions, number of excitations = 1, and the acquisition time was 16 min.

### Generalized q-sampling imaging

Based on the Fourier transform between the diffusion magnetic resonance (MR) signals and the diffusion displacement, a new relationship can be deduced by directly estimating the spin distribution function (SDF) from the diffusion MR signals. This relationship leads to a new reconstructed method called generalized q-sampling imaging. GQI can provide directional and quantitative information about crossing fibers.

GQI is a model-free method that quantifies the density of water, which diffuses in different orientations. Model-free methods estimate the empirical distribution of the water diffusion, and there is no hypothesis on the distribution. The SDF is the density of diffusing water in different directions and is a kind of diffusion orientation distribution function (ODF). GQI provides information of the relation between the diffusion signals of water and the SDF. GQI can be applied to grid or shell sampling schemes, q-ball imaging (QBI) and diffusion spectrum imaging (DSI). Studies have shown that GQI has good sensitivity and specificity for white matter properties and pathology [9].

The GQI indices included generalized fractional anisotropy (GFA), quantitative anisotropy (QA), normalized quantitative anisotropy (NQA) and the isotropic value of the orientation distribution function (ISO). GFA is defined as the standard deviation divided by the root mean square of the ODF, indicating a measurement of the anisotropy. QA is defined as the amount of anisotropic spins that diffuse along the fiber orientation. NQA is the normalized QA. ISO is the minimum distribution value of an ODF, and thus ISO represents the background isotropic diffusion [9].

### Graph theory

The human brain is a complex nervous system with highly segregated and integrated functions. We can construct a complex neural network model through the connections among brain regions. Graph theory is the mathematical study of graphs that model objects (“nodes”) and their connections (“edges”), where nodes represent brain regions and edges represent structural or functional connections between regions [10]. According to the related information, the brain network is divided into the white matter network and the gray matter network. The white matter network represents the connection of cerebral nerve fibers between brain regions, whereas the gray matter network represents the functional connectivity among brain regions.

Research on the white matter network of the brain by modern mathematical graph theory has proven that the structural network of the brain has the characteristics of a “small-worldness” topological structure, which means that it has high clustering of nodes and short path lengths between nodes [11]. The topology indices of graph theory include the mean clustering coefficient, gamma, local efficiency, characteristic path length, lambda, global efficiency and the small-worldness index. We used graph theoretical analysis and generalized q-sampling imaging to measure brain network connectivity.

### Image analysis

We used four methods, namely, voxel-based statistical analysis, graph theoretical analysis, network-based statistical analysis and multiple regression analysis, to analyze the diffusion data. We considered covariates in all the analyses.

### Voxel-based statistical analysis

Diffusion imaging was first corrected for eddy currents by FSL (FMRIB software library). The spin distribution function was reconstructed using a model-free reconstruction method with DSI studio (DSI studio was developed by Fang-Cheng (Frank) Yeh). Through this mathematical algorithm, we obtained the diffusion indices of generalized q-sampling imaging, including generalized fractional anisotropy (GFA), quantitative anisotropy (QA), normalized quantitative anisotropy (NQA) and the isotropic value of the orientation distribution function (ISO). Independent t-tests were performed with the Statistical Parametric Mapping (SPM) software to find the differences between the two groups. In addition, a significant difference in age between the two groups (*p* < 0.001) was found; thus, we considered age a covariate of no interest.

### Multiple regression analysis

In statistical modeling, regression analysis is a set of statistical processes for estimating the relationships among variables. It includes many techniques for modeling and analyzing several variables when the focus is on the relationship between a dependent variable and one or more independent variables. Multiple regression analysis is an extension of the application of simple linear regression that seeks to understand the function of a dependent variable and two or more sets of independent variables. Multiple regression analysis through SPM was used to detect the correlations between the neuropsychological scales and the indices of GQI for all participants. We also used age as a covariate in the multiple regression analysis.

### Graph theoretical analysis

Generalized q-sampling MRI can noninvasively detect the direction of water molecule diffusion in the white matter of the brain. We reconstructed the pathways of nerve fibers in the brain using fiber assignment by continuous tracking (FACT) with DSI studio. Network edges were established using FACT and the Automated Anatomical Labeling (AAL) templates, which divided the brain into 90 brain regions in Montreal Neurological Institute (MNI) space. The number of virtual fibers, or “edges”, connecting each pair of regions of interest (ROIs) was determined, resulting in a 90 × 90 weighted connectivity matrix for each participant [10]. We defined network edges as follows (1):

1 $$ \mathrm{E}=\frac{Fiber\ count}{Fiber\ length}\times NQA $$

Finally, the graph theoretical algorithm was used to obtain the topological properties of the complex network measures. The area under the curve (AUC) for each connectivity metric of the topology indices was compared between the groups. The network density range was calculated from 0.05 to 0.26, in 0.01 increments. The minimum value was defined by the limit density of the individual network not to be fragmented, and the maximum value was defined by the density when the topology indices that remained unchanged [12]. Since the differences between groups in network measures below the network density which depend on the number of individual networks that fragment in each group, group comparisons below the density are not meaningful [13]. The density means the ratio of existing connections to all possible connections. To identify the statistically significance differences between groups in the network topology indices, graph theoretical analysis toolbox was used to execute the two-sample *t*-test and non-parametric permutation test with 1000 repetitions. We evaluated the network segregation with the mean clustering coefficient, gamma, and local efficiency, and the network integration with the characteristic path length, lambda, and global efficiency [14].

### Network-based statistical analysis

Network-based statistic (NBS, Melbourne Neuropsychiatry Centre, The University of Melbourne and Melbourne Health, Australia) is the graph analogue of cluster-based statistical methods used in mass univariate testing on all pixels in an image. NBS analysis was used to identify the significance of any connected sub-networks obvious in the set of altered connections [15]. NBS analysis is used to identify any potentially connected structures formed by an appropriately chosen set of supra-threshold links. The topological extent of any such structure is used to examine its significance. The test statistic (i.e., primary threshold) computed for each pairwise combination is used to construct a set of supra-threshold links. The null distribution of the number of edges was empirically acquired using non-parametric permutation (5000 permutations) to evaluate the significance of each of the connected edges. Finally, we used the BrainNet viewer (The MathWorks Inc., Natick, MA, US) to visualize the significant sub-networks revealed by NBS.

## Results

A total of 39 participants were recruited for the study including 19 chemotherapy treated women and 20 healthy controls. All participants were aged between 20 and 55 years old. The average age of patients and healthy women were 43.8 ± 6.4 and 50.1 ± 2.5 years old. Due to wide range and significant difference between groups in age, we had added age as one of covariant factors in statistical analysis to reduce the impact of age. Two patients suffered from breast cancer stage I, 14 patients suffered from breast cancer stage II, and 3 patients suffered from breast cancer stage III, respectively. There were 4 patients received radiation therapy and 1 patient received hormonal treatment among 19 patients treated with chemotherapy. The number of menopausal women in the patients and controls were 5:5. The chemotherapy treated patients did not differ from the healthy controls with regard to education, MMSE, CAMS-R, HADS and IES-R. However, the breast cancer survivors showed significantly lower perceived cognitive impairments and impacts on quality of life, as revealed by paired t-tests (*p* < 0.05). The chemotherapy-treated patients revealed worse cognitive function. The participant demographic information and neuropsychological assessment results are presented in Table 1.

### Voxel-based statistical analysis

**Fig. 1:**
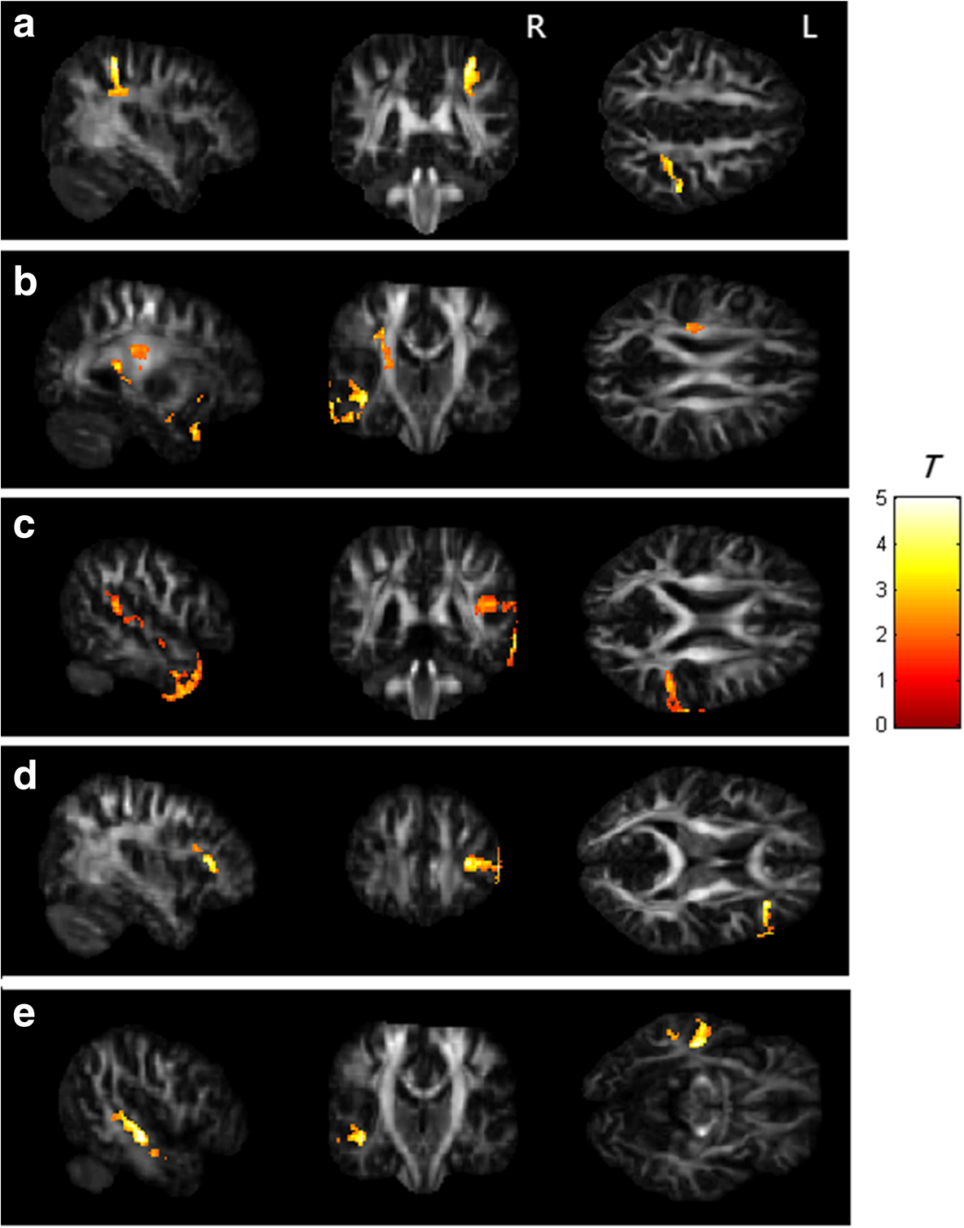
The GFA values of the breast cancer survivors were lower than those of the healthy controls in **a** the right postcentral blade, and lower NQA values were found in the brain regions of the **b** left superior corona radiate, **c** right superior temporal gyrus, **d** right inferior frontal blade and **e** left middle temporal gyrus

The results from the voxel-based statistical analysis showed significantly lower GFA and NQA values in the breast cancer group than those in the control group (*p* < 0.05 corrected by false discovery rate, FDR). The brain regions with differences included the right postcentral blade, left superior corona radiate, right superior temporal gyrus, right inferior frontal blade and left middle temporal gyrus. The results of the voxel-based statistical analysis are presented in Fig. 1.

### Multiple regression analysis

**Fig. 2:**
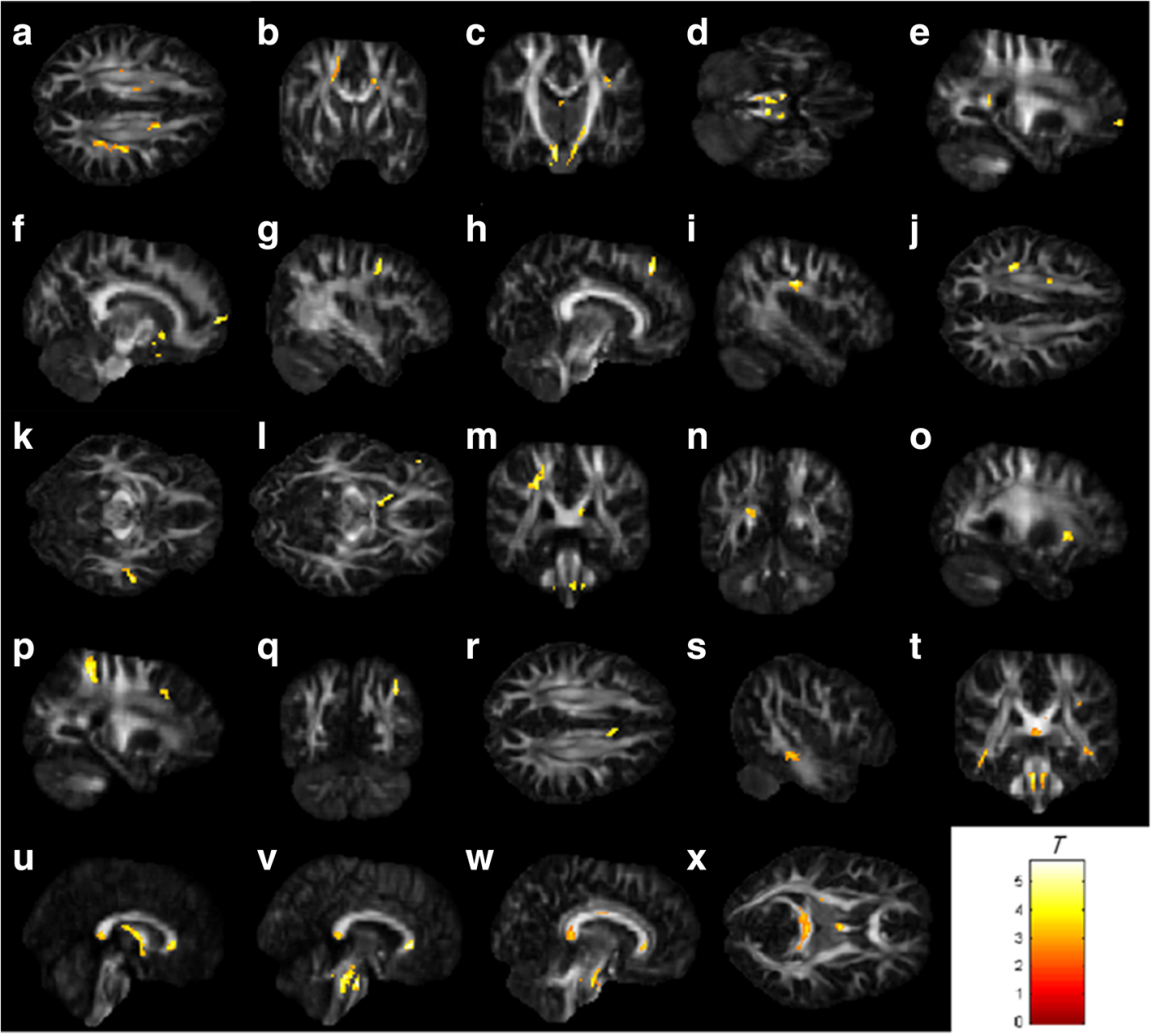
The results of the brain regions included in the multiple regression analysis. The brain regions (**a** to **x**) in Figure 2 correspond to the second column of Table 2

These neuropsychological scales (i.e., MMSE, CAMS-R, HADS, IES-R and FACT-Cog) were selected for the correlation analysis with changes in the indices of GQI. The results of the multiple regression analysis are shown in Table 2 and Fig. 2. It is worth mentioning that significant positive correlations between the perceived cognitive impairments and the GQI indices (GFA and NQA) were found in the regions of the left anterior corona radiate and the right cingulate gyrus (*p* < 0.01 corrected by FDR). In addition, significant positive correlations between the impact on quality of life and the GQI indices (GFA and NQA) were found in the regions of the right middle frontal gyrus, left postcentral blade and splenium of the corpus callosum (*p* < 0.01 corrected by FDR).

**Table 2:** Correlation between neuropsychological assessment scales and GQI indices

		MMSE	FACT-Cog			CAMS-R	IES-R	HADS	
			Perceived cognitive impairments	Impact on quality of life	Comments from others			Anxiety	Depression
Right superior corona radiata	a	☆							
Left superior corona radiata	b	☆				☆△			
Right cerebral peduncle	c	△							
Left cerebral peduncle	c	☆					★		
Right corticospinal tract	d	△							
Left corticospinal tract	d	☆△							
Right superior frontal gyrus	e	△							
Left superior frontal gyrus	f				△		▲		
Right middle frontal gyrus	g			☆△					
Left middle frontal gyrus	h	△				☆△			
Right superior longitudinal fasciculus	i	△							
Left superior longitudinal fasciculus	j					☆			
Right middle temporal gyrus	k					☆			
Left anterior limb of internal capsule	l						▲		
Left post-central blade	m			△					
Left precuneus	n				△				
Left anterior corona radiata	o		☆△		△				
Right superior parietal loblue	p								▲
Right inferior parietal loblue	q								▲
Right cingulate gyrus	r		☆△						
Sagittal stratum	s	☆							
Medial lemniscus	t	☆							
Column and body of fornix	u	△							
Pontine crossing tract	v	△							
Genu of corpus callosum	w	☆△							
Splenium of corpus callosum	x	☆△		△	△			★▲	

☆ = GFA(┼), ★ = GFA(—)

△ = NQA(┼), ▲ = NQA(—)

☆ means that GFA was positively correlated with neuropsychological assessment scales

★ means that GFA was negatively correlated with neuropsychological assessment scales

△ means that NQA was positively correlated with neuropsychological assessment scales

▲ means that NQA was negatively correlated with neuropsychological assessment scales

### Graph theoretical analysis

**Fig. 3:**
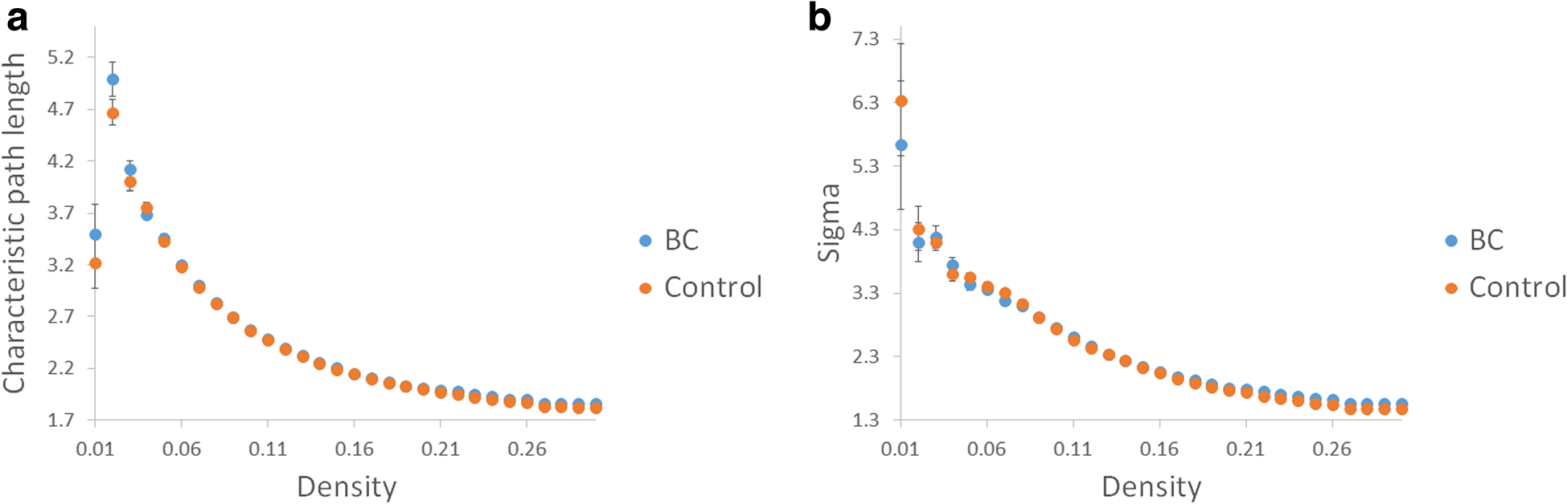
The breast cancer (BC) group (blue points) showed significantly longer characteristic path lengths in (**a**) than those of the controls (orange points), and both groups of women showed connectomes with the small-world properties of complex networks in (**b**)

In the graph theoretical analysis, we divided the individual topology network measurement into the BC and healthy control group. If the density was below 0.05, the individual network in both groups began to fragment which resulted in different numbers of nodes for individual network. Therefore, group comparisons below the density were not meaningful. The highest density was defined by the topology network measurement remained unchanged. The density we calculated is from 0.05 to 0.26. These results were confirmed by AUC analysis across network densities. The BC group demonstrated significantly longer characteristic path lengths across densities than those of the control group (*p* < 0.05 corrected by FDR, Fig. 3a). Longer characteristic path length represents worse global integration in the BC group. As shown in Fig. 3b, both groups of women demonstrated connectomes with the small-world properties (small-worldness index > 1) of complex networks when compared to random networks. Although both groups maintained the small-worldness brain network, the network was more like a regular network in the BC group.

### Network-based statistical analysis

**Fig. 4:**
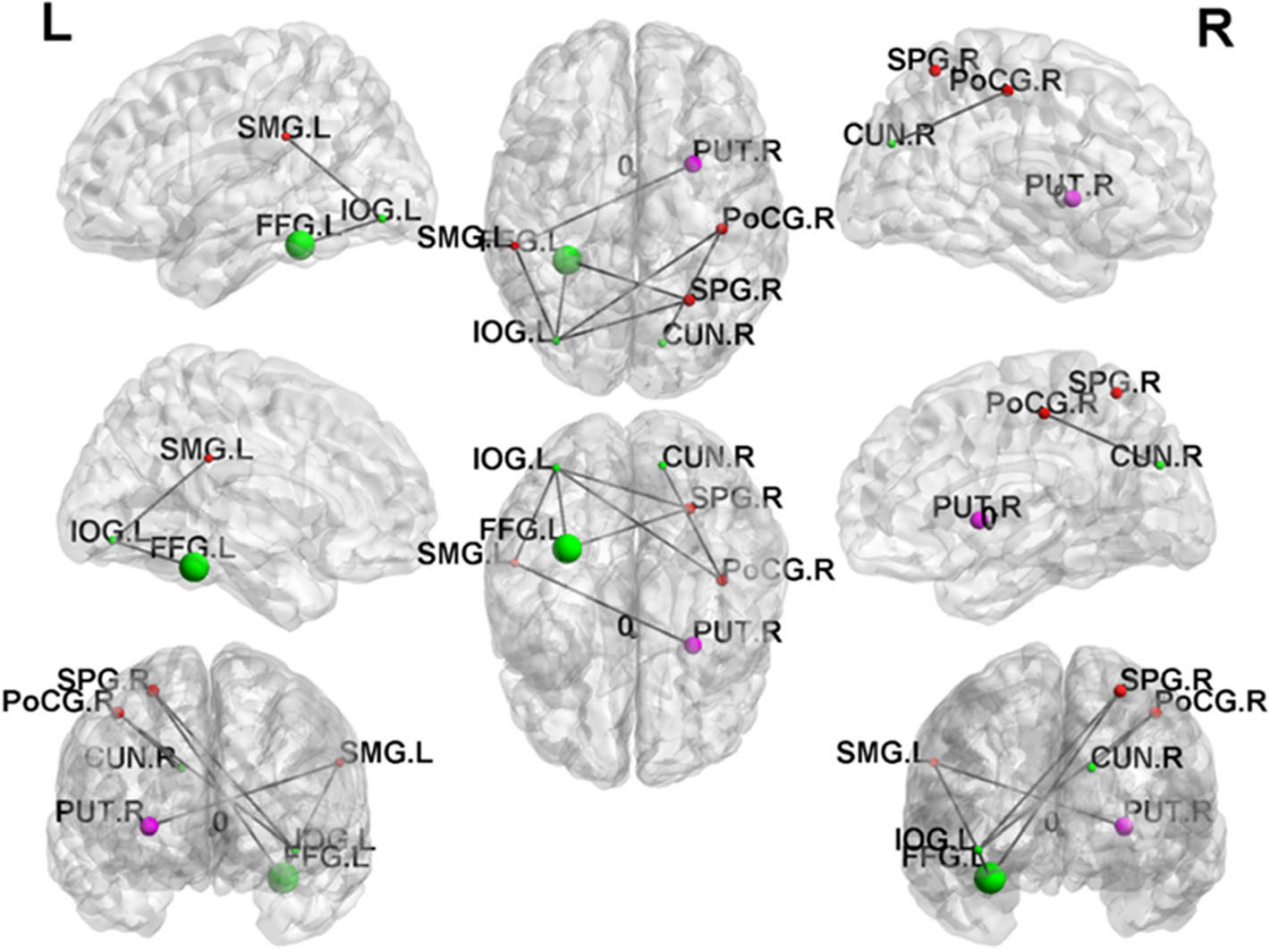
The brain structure network connections of the breast cancer group were decreased in the links between left inferior occipital and left fusiform, right post-central, right superior parietal, and left supra-marginal; between right cuneus and right post-central; between left fusiform and right superior parietal; between left supra-marginal and right putamen

In NBS analysis, we compared the edges of the brain networks between the BC group and the healthy controls. The results of the network-based statistics showed that some brain structure network connections were decreased in the breast cancer group compared to those in the control group (*p* < 0.01 corrected by FDR), as shown in Fig. 4. The lower connected sub-network comprised edges between left inferior occipital and left fusiform, right post-central, right superior parietal, and left supra-marginal; between right cuneus and right post-central; between left fusiform and right superior parietal; between left supra-marginal and right putamen.

## Discussion

Neuroimaging studies have shown that cognitive impairment results in subtle and diffuse brain damage [7, 16–18]. The cerebral white matter mediates communication among different brain regions, and the integrity of the cerebral white matter is important for the optimal performance of the brain. Injury to any part of the white matter connections can lead to changes in cognitive performance [19]. A study demonstrated that breast cancer survivors (3 to 5 months after chemotherapy) showed significantly worse performance in terms of attention, concentration, memory and psychomotor speed, as well as decreases in the FA of major white matter structures associated with cognitive function, such as the superior longitudinal fasciculus and the corpus callosum [20]. The control group showed higher scores on the FACT-Cog than those of the BC group, indicating that the cognitive function of breast cancer survivors decreased generally in our study. However, there were significant differences between the groups in perceived cognitive impairments and impact on quality of life. The effects of chemotherapy may lead to deficits in behavior and neuropsychological performance [21]. In our study, we did not find any significant differences between the groups in terms of the neuropsychological assessments, such as the MMSE, CAMS-R, HADS and IES-R.

The mechanisms mediating cognitive impairment after chemotherapy are unknown. There are several potential reasons for white matter vulnerability and cognitive function decline after chemotherapy, notably the direct white matter toxicity of chemotherapeutic drugs [7, 22]. There is evidence that the commonly used chemotherapeutic drug 5-fluorouracil (5-FU) crosses the blood brain barrier (BBB) by simple diffusion [23, 24]. Murine models have indicated that clinically relevant concentrations of 5-FU has been shown to cause injury to white matter tracts of the central nervous system; this finding was also reported in a case report on humans [25, 26]. Taxane-derived agents are chemotherapy drugs widely adopted in cancer treatment. Despite taxotere and epirubicin cross the BBB poorly [27], neurotoxicity is the major adverse effect of taxotere. They often manifested as painful neuropathy experienced during treatment, and it is sometimes irreversible [28]. There were 51 studies reported taxane-related gastrointestinal, hematological and neurological toxicities in adult patients with solid tumors [29]. In addition, epirubicin frequently induced toxicities including anemia, fever, myalgias, and neurotoxicity [30]. Therefore, we inferred that the effect of neurotoxicity was on the brain areas of the right post-central blade, left superior corona radiate, right superior temporal gyrus, right inferior frontal blade and left middle temporal gyrus. One study demonstrated decreased FA in the frontal and temporal white matter tracts of post-chemotherapy breast cancer patients compared to the tracts of healthy controls [31]. Another study showed decreased network connectivity in the frontal, striatal and temporal regions of cancer survivors 10 years after chemotherapy compared to that of healthy controls [32]. It has been demonstrated that medial temporal lobe toxicity has adverse effects on both recognition and working memory [33]. Our results were consistent with those of previous studies of the frontal and temporal regions.

A study examined the effect of adjuvant chemotherapy on white matter in women with breast cancer using DTI. It was found that positive correlation existed between the FA in the genu and processing speed [34]. Another study showed significant positive correlations between the domains of attention and processing/psychomotor speed and FA in the regions of the temporal and parietal white matter tracts. Moreover, the self-report cognitive failure questionnaire (CFQ) scores negatively correlated with the FA in the frontal and parietal regions [31]. We observed that the impact on quality of life was positively correlated with GFA and NQA in the splenium of the corpus callosum and the right middle frontal gyrus. Furthermore, the CAMS-R scores positively correlated with GFA in the right middle temporal gyrus and the left middle frontal gyrus. Our results were similar to those of previous studies.

The effect of menopause associated with cognitive function is uncertain [35]. Therefore, we did not consider menopause a covariate in this study. One study found no negative effects of therapy-induced menopause on cognitive function in breast cancer survivors [36]. Most studies divide breast cancer into 4 major molecular subtypes including Luminal A, Luminal B, Triple-negative, HER2 positive [37]. Whether breast cancer itself affects cognitive function is unknown. There is no literature to investigate this issue until now. Because the sample size is small and this is a preliminary research, we did not consider breast cancer subtypes in this study.

Cognitive deficits are common complications of breast cancer survivors treated with chemotherapy, and the incidence of cognitive deficits can reach 75% [38]. Chemotherapy-treated patients were found to be eight times more likely to have cognitive deficits than nonchemotherapy-treated patients [39, 40]. The most frequently observed disorders involve executive function and memory impairment. One study consisted of two groups of participants, including 34 breast cancer survivors who had completed chemotherapy more than 5.35 years before on average and 27 healthy women. The researchers explored changes in resting-state functional connectivity networks with graph theoretical analysis between breast cancer survivors treated with chemotherapy and healthy women. In addition, they evaluated the relationships among network measures, the length of time after chemotherapy, age, and breast cancer stage using linear regression analysis. The results showed that the clustering coefficient, characteristic path length and small-worldness index were lower in the breast cancer survivors than in the healthy controls. Compared with the control group, the breast cancer survivors had significantly lower nodal degree values in the left amygdala, left caudate, right inferior frontal gyrus, bilateral medial orbital frontal gyrus, and bilateral superior temporal gyrus. Linear regression analysis showed that the regional degree in the left hippocampus and right hippocampus were negatively correlated with the time since treatment. The impact of chemotherapy on the connectivity of these brain areas is permanent and may worsen over time [41].

Complex networks of the brain can be economical by minimizing the wiring cost, such as by possessing multiple nearby and fewer remote connections [42]. In our study, both groups of women demonstrated connectomes with the small-world properties of complex networks when compared to the properties of random networks. A small-worldness network has high local efficiency and global efficiency so that the brain network can effectively transfer information [43]. The human brain has been demonstrated to possess connectomes with the small-world properties that not only have the ability to segregate and integrate information [44] but also have low energy consumption and high efficiency in transmitting and processing information [42, 45].

This study used GQI and graph theoretical analysis to evaluate the brain structure and networks of chemotherapy-treated breast cancer survivors in comparison with controls. The results showed that the reduction in white matter connectivity in patients with breast cancer after treatment may lead to large-scale brain network reorganization, leading to increases in segregation and decreases in integration of the brain structural network. The changes in the small-world properties could reflect a compensatory mechanism, meaning the brain strives to maintain the integrity of the entire network at the expense of other networks, such as network integration [10]. Breast cancer survivors are usually able to perform a variety of cognitive tasks (intact segregation) but need more time, more effort or different strategies than before (damaged integration) [46, 47]. We found that the decreasing network integration in the breast cancer survivors was the result of the characteristic path length. The ability of the brain network is weak in transmitting messages. This finding was consistent with the concept that the white matter pathway plays a role in brain information transmission [48]. There were no significant differences between the groups in terms of network segregation in our study. Due to compensatory neuroplasticity, the cognitive function of breast cancer survivors may remain unchanged or may only slightly deteriorate [49].

### Limitations

The study was a preliminary study and more comprehensive investigations will be performed in the near future. There were some limitations in this study including small sample of participants, the cross-sectional design, and lack of a non-chemotherapy treated patient group. Therefore, we cannot distinguish the toxicity of chemotherapeutic drugs and the breast cancer itself on white matter structures. In addition, there was also variability in breast cancer stage, breast cancer subtypes, hormonal treatment, and menopause status that are likely to contribute to the effects of chemotherapy on cognitive function.

## Conclusion

Our results provide further evidence that adjuvant chemotherapy is associated with demyelination of white matter. In addition, adjuvant chemotherapy affected the integrity of white matter and resulted in poor cognitive performance, as indicated by the correlation between the neuropsychological assessment scales and the GQI indices. We found that the characteristic path lengths of breast cancer survivors were longer than those of healthy controls, as assessed by graph theoretical analysis. This result indicated that the brain network integration of breast cancer survivors became worse. Our study demonstrated alterations in the structural brain networks of breast cancer survivors. Therefore, changes in GQI indices and network topological properties may serve as neuropathological biomarkers of treatment-induced neurotoxicity. This is the first study to investigate chemotherapeutic effects on brain structural changes in breast cancer survivors with a generalized q-sampling image. Further studies of this issue with larger samples and longitudinal designs are required to determine the long-term effects of altered brain network organization.

## Abbreviations

5-FU: 5-fluorouracil
AAL: Automated Anatomical Labeling
AUC: Area under the curve
BBB: Blood brain barrier
BC: Breast cancer
CAMS-R: Cognitive and Affective Mindfulness Scale-Revised
CFQ: Cognitive failure questionnaire
CMF: Cyclophosphamide, methotrexate, and 5-flourouracil
CT: Chemotherapy
DSI: Diffusion spectrum imaging
DTI: Diffusion tensor imaging
FA: Fractional anisotropy
FACT: Fiber assignment by continuous tracking
FACT-Cog: Functional Assessment of Cancer Therapy-Cognitive Function
GFA: Generalized fractional anisotropy
GQI: Generalized q-sampling imaging
HADS: Hospital Anxiety and Depression Scale
IES-R: Impact of Event Scale-Revised
ISO: Isotropic value of the orientation distribution function
MD: Mean diffusivity
MMSE: Mini-Mental State Examination
MNI: Montreal Neurological Institute
MRI: Magnetic resonance imaging
NBS: Network-based statistic
NQA: Normalized quantitative anisotropy
ODF: Orientation distribution function
QA: Quantitative anisotropy
QBI: Q-ball imaging
RD: Radial diffusivity
ROI: Region of interest
RT: Radiotherapy
SDF: Spin distribution function
SPM: Statistical Parametric Mapping
